# Comparison of dyslipidemia incidence in Chinese early-stage breast cancer patients following different endocrine therapies: A population-based cohort study

**DOI:** 10.3389/fendo.2022.815960

**Published:** 2022-09-06

**Authors:** Junren Wang, Jin Yin, Jiajun Qiu, Jingwen Jiang, Yao Hu, Kunrui Zhu, Hong Zheng, Ting Luo, Xiaorong Zhong

**Affiliations:** ^1^ West China Biomedical Big Data Center, West China Hospital, Sichuan University, Chengdu, China; ^2^ Med-X Center for Informatics, Sichuan University, Chengdu, China; ^3^ School of Computer Science and Engineering, University of Electronic Science and Technology of China, Chengdu, China; ^4^ Cancer Center, Breast Disease Center, West China Hospital, Sichuan University, Chengdu, China; ^5^ Laboratory of Molecular Diagnosis of Cancer, Clinical Research Center for Breast, West China Hospital, Sichuan University, Chengdu, China

**Keywords:** breast cancer, dyslipidemia, endocrine therapy, cohort study, accumulated 5-year incidence

## Abstract

**Background:**

There is lack of large-scale real-world research evidence showing the impact of endocrine therapy on blood lipids in Chinese breast cancer patients, especially those with premenopausal breast cancer. Based on a large breast cancer cohort at West China Hospital, we aimed to compare the risk of dyslipidemia between premenopausal and postmenopausal women based on the endocrine therapy used.

**Methods:**

A total of 1,883 early-stage breast cancer (EBC) patients who received endocrine monotherapy [selective estrogen receptor modulator (SERM) and aromatase inhibitor (AI), with or without ovarian function suppression] with normal blood lipid levels at baseline were retrospectively included between October 2008 and April 2017. Dyslipidemia was defined as an abnormality in cholesterol, low-density lipoprotein cholesterol (LDL-C), high-density lipoprotein, and total cholesterol (TC) levels. The risk accumulation function was used to calculate the incidence of dyslipidemia in order to assess the absolute risk, while the multivariate Cox regression model was used to calculate the relative risk of dyslipidemia between the groups.

**Results:**

Patients with EBC were followed up for 60 months to monitor their blood lipid levels. The accumulated 5-year incidence of dyslipidemia in postmenopausal patients was higher than that in premenopausal patients (adjusted HR [95% confidence interval], 1.25 [1.01–1.56], 41.7% vs. 31.2%, *p* = 0.045). In premenopausal patients, the risk of abnormal TC was significantly higher in the OFS+AI group compared with that in the SERM group (adjusted HR [95% CI], 6.24 [3.19–12.20], *p* < 0.001, 5-year abnormal rates: 21.5% vs. 2.4%), and that of abnormal LDL-C level also increased (adjusted HR [95% CI], 10.54 [3.86–28.77], *p* < 0.001, 5-year abnormal rates: 11.1% vs. 0.9%). In postmenopausal patients, the risk of abnormal TC or LDL-C levels showed a similar trend in the AI and SERM groups.

**Conclusions:**

In addition to postmenopausal patients, dyslipidemia is also common in premenopausal Chinese patients with EBC who received endocrine therapy. Irrespective of menopausal status, AI treatment increases the risk of TC/LDL-C dyslipidemia than SERM treatment.

## Introduction

Breast cancer (BC) is the most common malignant tumor in Chinese women (1). Dyslipidemia, abnormal bone metabolism, mental anxiety, and depression are common concomitant diseases and are important components of the full-course management of BC (2). Dyslipidemia refers to an increase in serum cholesterol, triglyceride (TG), or low-density lipoprotein cholesterol (LDL-C) levels, or a decrease in high-density lipoprotein cholesterol (HDL-C) level (3), and is the primary risk factor for arteriosclerotic cardiovascular disease (ASCVD) occurrence and development (4, 5). According to the epidemiological evidence in China, increased cholesterol is the most important and clear risk factor for ASCVD, while high LDL-C is the third leading risk factor for CVD-related death in this country (6). Previous cohort studies have found that cardiovascular death accounts for approximately 16.3% of the total deaths among BC patients (7). Compared with BC patients without heart disease, those with heart disease have a 59% higher recurrence rate and a 60% higher mortality rate; moreover, myocardial infarction can accelerate BC progression and metastasis (8). For patients with BC, blood lipid management should be performed in the early stages to reduce the risk of developing heart disease.

Endocrine therapy is one of the standard treatments for estrogen receptor (ER)-positive BC and is mainly divided into selective estrogen receptor modulators (SERMs) and aromatase inhibitors (AIs) (9). Different endocrine drugs may have different effects on the blood lipid levels in patients with BC. Tamoxifen has good cardiovascular effects in postmenopausal BC patients, including reduced total cholesterol and LDL-C levels and increased HDL-C levels (10, 11). The ATAC trial compared the adverse reactions of anastrozole and tamoxifen in postmenopausal patients with BC, and the results of a 100-month follow-up showed that the incidence of hypercholesterolemia in patients treated with anastrozole was significantly higher than that in patients treated with tamoxifen (12). In a previous prospective clinical trial, Shien et al. found that TC and LDL-C levels at 12 and 24 months after toremifene treatment were lower than those in the letrozole group (13). A retrospective study found that the level of TGs in postmenopausal BC patients treated with letrozole for 24 months was higher than that in patients treated with exemestane (14).

Therefore, monitoring for dyslipidemia is of utmost importance. However, there is lack of large-scale real-world research evidence on the impact of different endocrine therapies on blood lipids in Chinese BC patients, especially in premenopausal BC patients. Based on a large cohort of BC patients in West China Hospital of Sichuan University, we aimed to compare the effects of different endocrine therapies, SERMs, and AIs, on the blood lipid levels in premenopausal and postmenopausal women.

## Methods

### West China Hospital breast cancer cohort

Patients pathologically diagnosed with BC have been prospectively registered in the Breast Cancer Information Management System at West China Hospital, Sichuan University since 2008 (15–18). Oncologists obtained the patients’ medical records, diagnostic pathology reports, and treatment data. All patients were followed up through outpatient visits or a telephone call at 3- to 4-month intervals within 3 years after diagnosis, 6-month intervals within 4–5 years, and then annually. This study was approved by the Biomedical Research Ethics Committee of West China Hospital (reference number: 20200427).

### Study design

**Figure 1:**
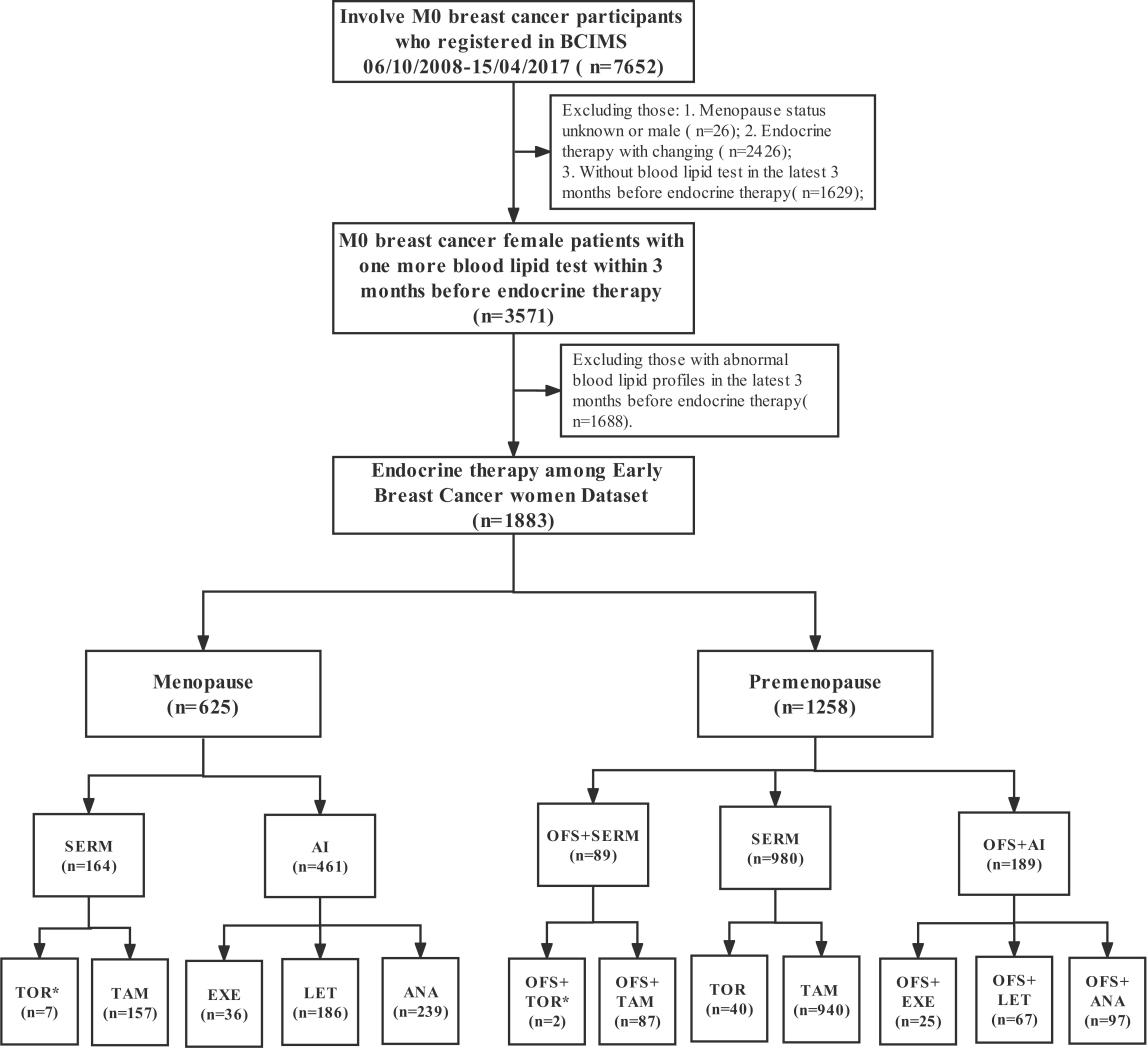
The flowchart shows the participant selection process. *As the number of participants were relatively small, some drugs were not included in the cumulative dyslipidemia incidence analysis of different endocrine therapy drugs.

In this study, 7,652 early-stage breast cancer (EBC) patients from the WHC BC cohort with pathologically confirmed non-metastatic disease at the time of diagnosis were retrospectively enrolled from 6 October 2008 to 15 April 2017. We excluded 9 patients with unknown menopause status and 17 male patients. To avoid interference with the analysis by the endocrine conversion regimen, only patients receiving one endocrine therapy regimen were included, while 2,426 patients receiving multiple SERM or AI during the endocrine adjuvant therapy phase were excluded. We also excluded 3,317 patients with unknown lipid status or dyslipidemia in the last 3 months before the initiation of endocrine therapy. Overall, only 1,883 women with EBC were included in this study. A flowchart of the study selection process is presented in **Figure 1**.

### Definition of dyslipidemia

Blood lipid test data were obtained using the laboratory information management system at the West China Hospital of Sichuan University. According to the “2016 Chinese guideline for the management of dyslipidemia in adults,” (3) any of the following conditions indicates dyslipidemia: total cholesterol of ≥6.2 mmol/L, LDL of ≥4.1 mmol/L, HDL of <1.0 mmol/L, or TG of ≥2.3 mmol/L. Since the above indexes have different physiological meanings, the outcome of our interest was defined as abnormalities in the total cholesterol, LDL-C, HDL-C, and TG levels during 1–60 months after receiving endocrine therapy. To avoid the underestimation of follow-up time, the end point of follow-up, for patients with dyslipidemia during 1–60 months, was defined as the last time to receive a lipid test or 60 months.

### Covariates

The demographic characteristics (age and BMI) and clinical information (menstrual status, disease stage, ER, progesterone receptor, human epidermal growth factor receptor 2, endocrine therapy, etc.) of the WHC BC cohort were collected. Data on the history of treatment with lipid-lowering drugs were extracted from the electronic medical records. The different SERMs used for endocrine therapy included tamoxifen (TAM) and toremifene (TOR). AIs used included anastrozole (ANA), letrozole (LET), and exemestane (EXE). Ovarian function suppression (OFS) involved goserelin drug castration or bilateral ovariectomy surgical castration. Comorbidity was any history of hypertension, diabetes, and chronic kidney disease.

### Data analysis

Descriptive statistics were obtained for all the study variables. Continuous variables were expressed as mean (standard deviation) or median (interquartile range [IQR]) values. Categorical data were expressed as numbers (proportions). A cumulative incidence analysis and log-rank test were used to assess the cumulative incidence rates of dyslipidemia, which was used to assess the absolute risk of dyslipidemia within 60 months in patients treated with different endocrine therapies. Pairwise comparisons between multiple groups were carried out using the log-rank test, and the *p*-value was adjusted using the Benjamini–Hochberg procedure. Multivariate Cox regression models, involving variables with a *p*-value of less than 0.05, were used to assess the relative risk for dyslipidemia in different groups. The relative risk of dyslipidemia within 5 years was assessed in premenopausal and postmenopausal patients treated with different endocrine drugs. Data analysis and graph drawing were performed using R version 3.6.2. Statistical analyses were performed using Microsoft Excel 2016 (Microsoft). For all statistical analyses, a *p*-value of <0.05 was considered significant.

## Results

### Participants’ characteristics

**Figure 2:**
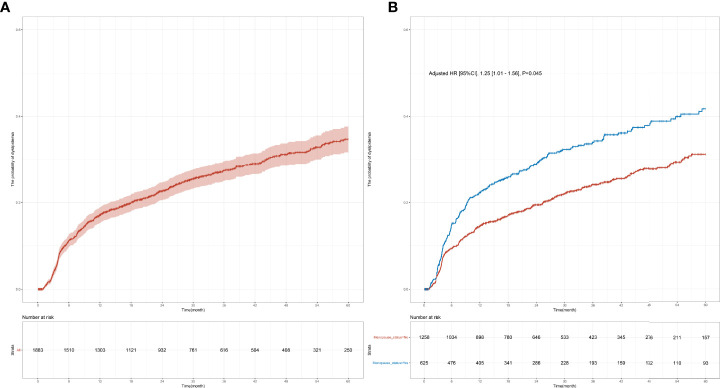
Cumulative dyslipidemia incidence among premenopausal and menopausal early-stage breast cancer patients (EBC). Cumulative curves **(A)** showing dyslipidemia incidence in all EBC patients receiving endocrine therapy and **(B)** showing dyslipidemia incidence among different menopausal groups.

In this study, 1,883 female patients with normal blood lipid levels prior to the initiation of endocrine therapy were followed up for 60 months to monitor their blood lipid levels. Among them, 1,258 were premenopausal women, the median age was 43.00 years (IQR, 39.00–47.00), and the median BMI was 22.22 (IQR, 20.45–24.14); among the premenopausal women, 298 had dyslipidemia within 60 months, and the median follow-up time was 31.58 months (IQR, 0.03–60.00). Meanwhile, 625 participants were postmenopausal patients, the median age was 57.00 years (IQR, 52.00–62.00), and the median BMI was 23.44 (IQR, 21.23–25.63); among postmenopausal women, 207 had dyslipidemia within 60 months, and the median follow-up time was 31.79 months (IQR, 0.13–60.00). The incidence of dyslipidemia within 1 year was significantly higher in postmenopausal patients compared with that in premenopausal patients (22.3% [25.6%–18.8%] vs. 14.6% [16.7%–12.6%], *p* < 0.001; **Table 1**). The 5-year risk of dyslipidemia was also significantly higher in postmenopausal patients compared with that in premenopausal patients (adjusted hazard ratio (HR) [95% confidence interval (CI)], 1.25 [1.01–1.56], *p* = 0.045; 41.7% vs. 31.2%%, *p* < 0.001; **Table 1** and **Figure 2**) after adjusting for variables that were significant in the univariate Cox regression (i.e., age, BMI, stage, comorbidity, and KI67). Furthermore, the time of dyslipidemia onset was extremely close between postmenopausal and premenopausal patients (7.45 months [1.06–59.23] vs. 8.35 months [1.03–57.00]; **Table 1**).

**Table 1:** Characteristics and dyslipidemia risk among early-stage breast cancer patients.

			Menopausal status		
	Group	All (*n* = 1,883)	Postmenopause (*n* = 625)	Premenopause (*n* = 1,258)
Age, median (IQR)		46.00 [41.00–53.00]	57.00 [52.00–62.00]	43.00 [39.00–47.00]
		47.31 (9.63)	57.42 (7.18)	42.28 (6.10)
BMI, median (IQR)		22.58 [20.70–24.69]	23.44 [21.23–25.63]	22.22 [20.45–24.14]
		22.88 (3.02)	23.63 (3.16)	22.51 (2.88)
Stage, *n* (%)	0	36 (1.9)	11 (1.8%)	25 (2.0%)
	I	433 (23.0)	141 (22.6%)	292 (23.2%)
	II	887 (47.1)	297 (47.5%)	590 (46.9%)
	III	431 (22.9)	159 (25.4%)	272 (21.6%)
	Unknown	96 (5.1)	17 (2.7%)	79 (6.3%)
pT status, *n* (%)	0	45 (2.4)	17 (2.7%)	28 (2.2%)
	1	668 (35.5)	229 (36.6%)	439 (34.9%)
	2	891 (47.3)	307 (49.1%)	584 (46.4%)
	3	79 (4.2)	18 (2.9%)	61 (4.8%)
	4	109 (5.8)	43 (6.9%)	66 (5.2%)
	Unknown	91 (4.8)	11 (1.8%)	80 (6.4%)
pM status, *n* (%)	0	1,883 (100.0)	625 (100.0%)	1,258 (100.0%)
pN status, *n* (%)	0	927 (49.2)	293 (46.9%)	634 (50.4%)
	1	593 (31.5)	196 (31.4%)	397 (31.6%)
	2	207 (11.0)	78 (12.5%)	129 (10.3%)
	3	144 (7.6)	51 (8.2%)	93 (7.4%)
	Unknown	12 (0.6)	7 (1.1%)	5 (0.4%)
Subtype, *n* (%)	Luminal A	297 (15.8)	83 (13.3%)	214 (17.0%)
	Luminal B (Her2 +)	299 (15.9)	91 (14.6%)	208 (16.5%)
	Luminal B \(Her2 −)	752 (39.9)	237 (37.9%)	515 (40.9%)
	TNBC	22 (1.2)	12 (1.9%)	10 (0.8%)
	Her2 positive	25 (1.3)	10 (1.6%)	15 (1.2%)
	Unknown	488 (25.9)	192 (30.7%)	296 (23.5%)
ER, *n* (%)	Negative	139 (7.4)	67 (10.7%)	72 (5.7%)
	Positive	1,714 (91.0)	543 (86.9%)	1,171 (93.1%)
	Unknown	30 (1.6)	15 (2.4%)	15 (1.2%)
PR, *n* (%)	Negative	253 (13.4)	139 (22.2%)	114 (9.1%)
	Positive	1,599 (84.9)	471 (75.4%)	1,128 (89.7%)
	Unknown	31 (1.6)	15 (2.4%)	16 (1.3%)
HER2, *n* (%)	Negative	1,198 (63.6)	384 (61.4%)	814 (64.7%)
	Positive	357 (19.0)	118 (18.9%)	239 (19.0%)
	Unknown	328 (17.4)	123 (19.7%)	205 (16.3%)
KI67, *n* (%)	<14%	418 (22.2)	128 (20.5%)	290 (23.1%)
	≥14%	1,369 (72.7)	460 (73.6%)	909 (72.3%)
	Unknown	96 (5.1)	37 (5.9%)	59 (4.7%)
Comorbidity **^a^**, *n* (%)	No	1,669 (88.6)	475 (76.0%)	1,194 (95.0%)
	Yes	214 (11.4)	150 (24.0%)	64 (5.1%)
TC (mmol/L), median (IQR)		4.82 [4.29–5.33]	4.98 [4.44–5.45]	4.74 [4.22–5.25]
Mean (SD)		4.79 (0.72)	4.92 (0.71)	4.72 (0.72)
TG (mmol/L), median (IQR)		1.33 [1.01–1.66]	1.42 [1.09–1.72]	1.28 [0.97–1.64]
Mean (SD)		1.35 (0.43)	1.42 (0.42)	1.32 (0.43)
HDL-C (mmol/L), median (IQR)		1.43 [1.25–1.67]	1.41 [1.23–1.65]	1.45 [1.27–1.68]
Mean (SD)		1.49 (0.32)	1.47 (0.31)	1.50 (0.32)
LDL-C (mmol/L), median (IQR)		2.80 [2.37–3.23]	2.93 [2.50–3.37]	2.75 [2.31–3.16]
Mean (SD)		2.79 (0.61)	2.91 (0.61)	2.73 (0.60)
Follow-up (months), median (range)		31.68 [0.03–60.00]	31.79 [0.13–60.00]	31.58 [0.03–60.00]
No. of events within 5 years		467	193	274
Durations from baseline to dyslipidemia (months), median (range)		7.77 [1.03–59.23]	7.45 [1.06–59.23]	8.35 [1.03–57.00]
Incidence within 1 year [95% CI]		17.2% [18.9%–15.4%]	22.3% [25.6%–18.8%]	14.6% [16.7%–12.6%]
Incidence within 5 years [95% CI]		34.7% [37.7%–31.7%]	41.7% [46.7%–36.3%]	31.2% [34.7%–27.5%]

^a^Comorbidity for hypertension, diabetes, and chronic kidney disease; ER, estrogen receptor; PR, progesterone receptor; HER2, human epidermal growth factor receptor-2; TNBC, triple-negative breast cancer; TC, total cholesterol; TG, triglyceride; HDL-C, HDL cholesterol; LDL-C, LDL cholesterol.

### Abnormalities in four blood lipid indexes among premenopausal women with EBC

**Figure 3:**
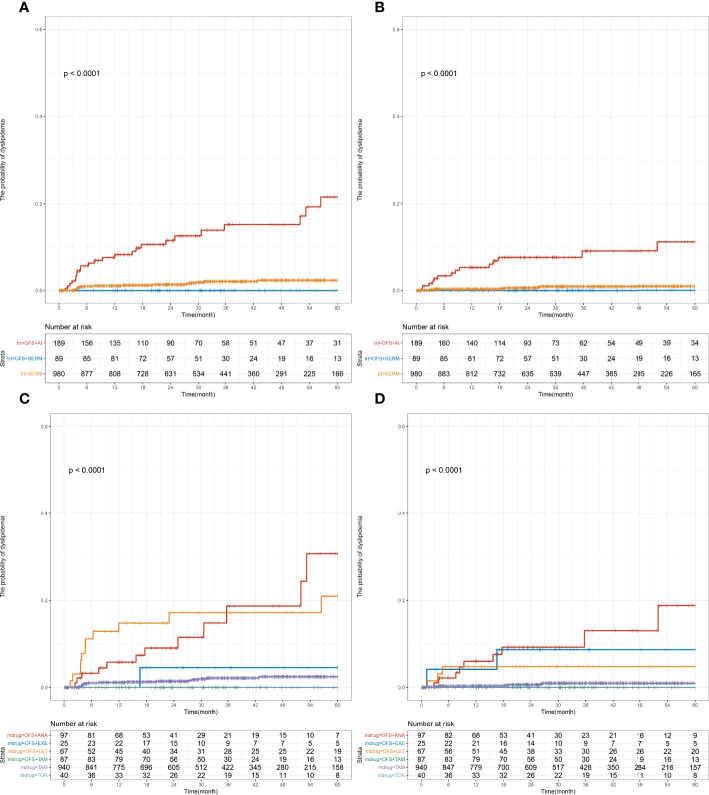
Cumulative incidence of abnormal TC and LDL-C levels among early-stage breast cancer premenopausal patients based on the endocrine therapy used. Cumulative curves showing TC **(A, C)** and LDL-C **(B, D)** dyslipidemia incidences. *p*-values were obtained from the log-rank tests for comparisons of abnormal TC and LDL-C incidences between the different endocrine therapy groups. SERM, selective estrogen receptor modulator; TAM, tamoxifen; TOR, toremifene; AI, aromatase inhibitor; ANA, anastrozole; LET, letrozole; EXE, exemestane; OFS, ovarian function suppression; TC, total cholesterol; TG, triglyceride; HDL-C, HDL cholesterol; LDL-C, LDL cholesterol.

The effect of specific endocrine therapy on the incidence of a single index of dyslipidemia was further analyzed. The premenopausal group received three types of endocrine therapies: SERM (77.9%), OFS+AI (15.0%), and OFS+SERM (7.1%). The specific treatment plans were as follows: TAM (74.7%) and TOR (3.2%), OFS+TAM (6.9%) and OFS+TOR (0.2%), OFS+ANA (7.7%), OFS+LET (5.3%), and OFS+EXE (2.0%). Prior to the initiation of endocrine therapy, the levels of the following blood lipids were measured: TC (4.74 [4.22–5.25] mmol/L), TG (1.28 [0.97–1.64] mmol/L), HDL-C (1.45 [1.27–1.68] mmol/L), and LDL-C (2.75 [2.31–3.16] mmol/L). Significant differences were observed in the risks of abnormal TC and LDL-C levels among the three groups (TC, *p* < 0.001; LDL-C, *p* < 0.001; **Figure 3**). Results of the pairwise log-rank test showed that the 1-year incidence (TC: OFS+AI vs. SERM, 8.2% vs. 1.1%, *p* < 0.001; LDL-C: OFS+AI vs. SERM, 5.3% vs. 0.3%, *p* < 0.001; **Supplementary Tables S1, 3**) and 5-year incidence of abnormal TC and LDL-C levels (TC: OFS+AI vs. SERM, 21.5% vs. 2.4%, *p* < 0.001; LDL-C: OFS+AI vs. SERM, 11.1% vs. 0.9%, *p* < 0.001; **Supplementary Tables S1, 3**) in the OFS+AI group were significantly higher than those in the SERM group. In the subgroup analysis of specific drugs, the 5-year incidence of TC and LDL-C in the OFS+ANA or OFS+LET group was significantly higher than that in the TAM group, but the difference between OFS+ANA and OFS+LET was not significant (**Supplementary Tables S1, 5**).

In order to determine the relative risk of using different treatment modes or drugs in patients with abnormal TC and LDL-C levels within a follow-up of 5 years, a multivariate Cox regression model was constructed, and the factors with significant single factor Cox regression *p*-values between the corresponding groups were included for correction; results showed that the OFS+AI group had a higher risk of developing abnormal LDL-C levels (adjusted HR [95% CI], 10.54 [3.86–28.77], *p* < 0.001) and abnormal TC levels (adjusted HR [95% CI], 6.24 [3.19–12.20], *p* < 0.001) than the SERM group. Compared with the TAM group, the OFS+ANA or OFS+LET group had a higher risk of developing abnormal LDL-C levels (OFS+ANA vs. TAM: adjusted HR [95% CI], 15.95 [5.30–48.02], *p* < 0.001; OFS+LET vs. TAM: adjusted HR [95%CI], 6.96 [1.68–28.78], *p* = 0.007) and abnormal TC levels (OFS+ANA vs. TAM: adjusted HR [95% CI], 6.30 [2.80–14.16], *p* < 0.001; OFS+LET vs. TAM: adjusted HR [95% CI], 8.15 [3.65–18.15], *p* < 0.001); other details are shown in **Table 2**. However, no statistical difference was found in the TG and HDL-C levels between the different endocrine therapy groups among premenopausal patients (**Supplementary Figure S1**).

**Table 2:** Hazard ratio of LDL-C and TC dyslipidemia among early-stage breast cancer patients based on endocrine therapy used.

Menopause	LDL-C				TC			
	Crude HR [95% CI]	P	Adjusted HR* [95% CI]	*p*	Crude HR [95% CI]	*p*	Adjusted HR* [95% CI]	*p*
AI vs. SERM	–	–	–	–	4.12 [1.90–8.94]	<0.001	3.94 [1.81–8.56]	<0.001
ANA vs. TAM	–	–	–	–	5.58 [2.38–13.04]	<0.001	5.32 [2.25–12.55]	<0.001
LET vs. TAM	–	–	–	–	3.88 [1.60–9.43]	0.003	3.58 [1.47–8.72]	0.005
ANA vs. LET	1.30 [0.70–2.41]	0.407	1.09 [0.58–2.06]	0.782	1.44 [0.89–2.33]	0.135	1.41 [0.87–2.29]	0.164
Pre-menopause	LDL-C				TC			
	Crude HR [95% CI]	*p*	Adjusted HR* [95% CI]	*p*	Crude HR [95% CI]	*p*	Adjusted HR* [95% CI]	*p*
OFS+AI vs. SERM	11.65 [4.70–28.89]	<0.001	10.54 [3.86–28.77]	<0.001	8.44 [4.53–15.74]	<0.001	6.24 [3.19–12.20]	<0.001
OFS+ANA vs. TAM	15.65 [5.80–42.24]	<0.001	15.95 [5.30–48.02]	<0.001	8.92 [4.23–18.78]	<0.001	6.30 [2.80–14.16]	<0.001
OFS+LET vs. TAM	6.32 [1.63–24.45]	0.008	6.96 [1.68–28.78]	0.007	9.86 [4.61–21.09]	<0.001	8.15 [3.65–18.15]	<0.001

SERM, selective estrogen receptor modulator; TAM, tamoxifen; TOR, toremifene; AI, aromatase inhibitor; ANA, anastrozole; LET, letrozole; EXE, exemestane; OFS, ovarian function suppression; TC, total cholesterol; LDL-C, LDL cholesterol; *adjusted for age, BMI, stage, and comorbidity. Refers to the unavailability of computing the HR for no dyslipidemia events occurring among menopausal patients receiving SERM (i.e., TAM).

### Abnormalities in four blood lipid indexes among postmenopausal women with EBC

**Figure 4:**
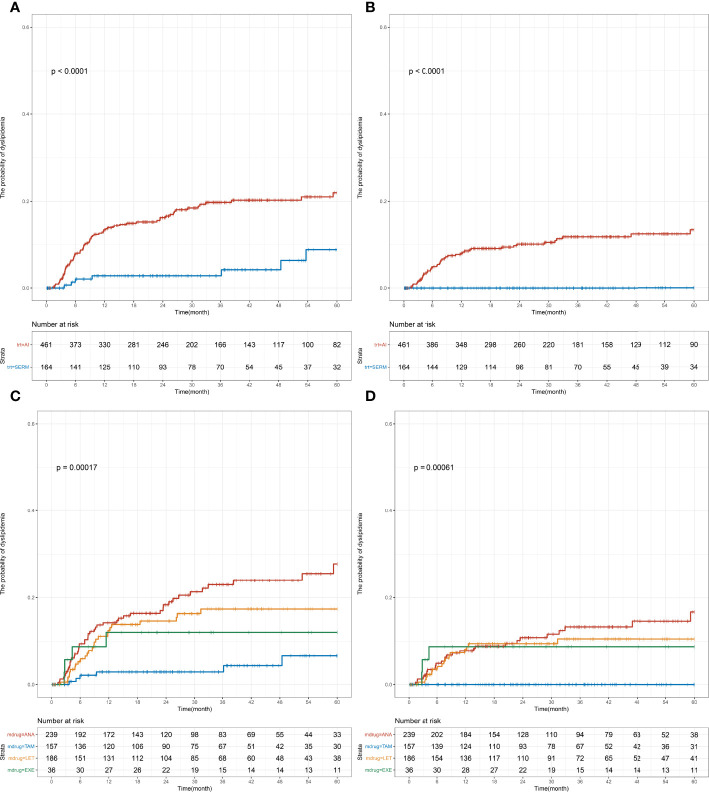
Cumulative abnormal TC and LDL-C incidence among early-stage breast cancer menopausal patients based on endocrine therapy used. Cumulative curves showing TC **(A, C)** and LDL-C **(B, D)** dyslipidemia incidences. *p*-values were obtained from the log-rank tests for comparisons of abnormal TC and LDL-C incidences between the different endocrine therapy groups. SERM, selective estrogen receptor modulator; TAM, tamoxifen; TOR, toremifene; AI, aromatase inhibitor; ANA, anastrozole; LET, letrozole; EXE, exemestane; TC, total cholesterol; TG, triglyceride; HDL-C, HDL cholesterol; LDL-C, LDL cholesterol.

In the postmenopausal group, SERM (26.2%) and AI (73.8%) were administered as endocrine therapy; the specific therapeutic drugs used were as follows: TAM (25.1%), TOR (1.1%), ANA (38.2%), LET (29.8%), and EXE (5.8%). Prior to the initiation of endocrine therapy, the following blood lipid levels were measured and the results were as follows: TC = 4.98 (4.44–5.45) mmol/L, TG = 1.42 (1.09–1.72) mmol/L, HDL-C = 1.41 (1.23–1.65) mmol/L, and LDL-C = 2.93 (2.50–3.37) mmol/L. Similar to the premenopausal group, significant differences were found in the risk of developing abnormal TC and LDL-C levels between the SERM group and AI group (TC: *p* < 0.001, LDL-C: *p* < 0.001; **Figure 4**). The 1-year incidence (AI vs. SERM, 13.3% vs. 2.8%, *p* = 0.003; **Supplementary Tables S2, 4**) and 5-year incidence of abnormal TC levels were higher in the AI group compared with that in the SERM group (AI vs. SERM, 22.0% vs. 8.8%, *p* < 0.001; **Supplementary Tables S2, 4**). Compared with the SERM group, the 1-year incidence of abnormal LDL-C levels in the AI group did not increase significantly (AI vs. SERM, 7.9% vs. 0.0%, *p* = 0.045), but the 5-year incidence of abnormal LDL-C levels increased significantly (AI vs. SERM, 13.3% vs. 0.0%, *p* < 0.001; **Supplementary Tables S2, 4**). In the subgroup analysis of specific drugs, the same trend was observed in the postmenopausal patients: the 5-year incidence of abnormal TC and LDL-C levels was significantly higher in the ANA or LET group compared with that in the TAM group, but the difference between ANA and LET was not significant (**Supplementary Table S6**).

Multivariate Cox regression model showed that the AI group had a higher risk of developing abnormal LDL-C levels (no applicable HR for the SERM group without dyslipidemia) and abnormal TC levels (adjusted HR [95% CI], 3.94 [1.81–8.56], *p* < 0.001; **Table 2**) than the SERM group. Compared with the TAM group, the ANA or LET group had a higher risk of developing abnormal LDL-C levels (no applicable HR for the TAM group without dyslipidemia) and abnormal TC levels (ANA vs. TAM: adjusted HR [95% CI], 5.32 [2.25–12.55], *p* < 0.001; LET vs. TAM: adjusted HR [95% CI], 3.58 [1.47–8.72], *p* = 0.005). No statistical difference was found in the TG and HDL-C levels between the different endocrine therapy groups among postmenopausal patients (**Supplementary Figure S2**).

## Discussion

Based on a large cohort, the present real-world study provided a comprehensive dyslipidemia profile following different endocrine therapies in not only postmenopausal but also premenopausal patients with EBC in China. Another strength of this study was the longer follow-up period of 5 years, which enabled the long-term evaluation of the changes in blood lipid levels. The risk of dyslipidemia in postmenopausal patients was higher than that in premenopausal patients. Furthermore, the dyslipidemia risk was compared among different groups of Chinese premenopausal women. The risk of developing abnormal TC and LDL-C levels in the OFS+AI group at 1 year or 5 years was significantly higher than that in the SERM group. Among postmenopausal patients, the 1-year or 5-year risk of developing abnormal TC levels in the AI group was significantly higher than that in the SERM group, and the 5-year risk of abnormal LDL-C in the AI group was also significantly higher than that in the SERM group.

At present, there is sufficient evidence regarding the risk factors for dyslipidemia in patients with BC. Li et al. conducted a survey in 5,375 people in Chongqing, China, and found that high BMI and age were independent risk factors for female dyslipidemia (19). Several epidemiological studies have shown that the dyslipidemia level in postmenopausal women is significantly higher than that in premenopausal women (20). In general, premenopausal women show lower atherogenic lipid profiles (higher HDL and antiatherogenic HDL2 levels and lower TG levels); after menopause, HDL decreases rapidly, while TG and LDL levels increase (21, 22). Therefore, the incidence of coronary heart disease increases with the onset of menopause and changes in the blood lipid levels (23). AIs significantly reduce systemic aromatization, inhibit estrogen synthesis, and reduce estrogen availability in various organs and tissues, such as the ovaries, breasts, adipose tissues, and musculoskeletal organs (23). Thus, the ability of estrogen to coordinate lipid and lipoprotein metabolism is further weakened by AIs. Hence, physicians should provide effective management for dyslipidemia in BC patients, especially those undergoing endocrine therapy after menopause.

In China, full-course management of BC and its associated diseases is receiving increasing research attention (2). However, the time of occurrence and incidence of dyslipidemia in hormone-positive EBC patients, especially in premenopausal patients receiving different endocrine therapies, remain unclear. This study found that after endocrine therapy, the 5-year incidence of dyslipidemia in postmenopausal patients was higher than that in pre-menopausal patients (postmenopausal vs. pre-menopausal patients: 42.6% vs. 32.6%). Among premenopausal BC patients, the OFS+AI group had a significantly higher risk of developing abnormal TC and LDL-C levels than the SERM group, while the OFS+LET or OFS+ANA group had a significantly higher risk of developing abnormal TC and LDL-C levels than the TAM group at 1 year or 5 years. For postmenopausal women with EBC, the risk of abnormal TC and LDL-C was also higher in the AI group than in the SERM group, which was consistent with the clinical reports of early hormone receptor-positive BC patients treated with endocrine therapy (13, 24–26). This finding suggests that more than one-third of premenopausal and postmenopausal women with BC may have dyslipidemia after receiving long-term endocrine therapy; moreover, SERM is more likely to cause changes in TC and LDL-C levels than AI, which are the two important risk factors of CVD.

Because the physiological meaning of blood lipid indicators differs, the impacts of different endocrine therapies were analyzed to adequately understand how different endocrine therapies affect the blood lipid profiles. Because of its ability to promote reverse cholesterol transport and improve atherosclerotic vascular lesions, HDL-C is often considered as a good cholesterol (27). Several epidemiological studies have reported a positive association between increased HDL-C serum levels and decreased CVD risk (27, 28). On the contrary, high LDL-C levels are significant risk factors for CVD (29). According to epidemiological evidence in China, increased cholesterol is the most important and clear risk factor for ASCVD in China, while increased LDL-C is the third largest risk factor for CVD-related death in China (6). After receiving endocrine therapy for 5 years, about one-third of patients with hormone receptor-positive EBCs developed dyslipidemia. This result suggests that dyslipidemia is one of the most common concomitant diseases associated with BC. In our study, we also found that female patients with EBC, whether postmenopausal or premenopausal, were significantly more likely to develop abnormal LDL-C and TC levels within 60 months of taking SERM drugs than those taking AI drugs. In light of the above findings, lipid profiles (especially LDL-C and TC) should be continuously monitored during the course of endocrine therapy, TAM should be used as first-line treatment in patients with cardiovascular risk factors, and statins should be used in female BC patients with high cholesterol levels.

Studies on the effects of AIs on lipid profiles remain controversial. Previous studies have suggested that letrozole and anastrozole are more likely to cause elevated cholesterol levels (30, 31). A subsequent phase III clinical trial also suggested that different AIs had different effects on lipid metabolism: TG and cholesterol levels increased significantly in the anastrozole group compared with that in the exemestane group (32). By contrast, the lipid profiles vary in patients using different AI treatments (14, 33). Our study showed higher risks of developing abnormal LDL-C and TC levels in the AI group compared with that in the SERM group among postmenopausal BC patients, but no difference was observed in the TG and HDL-C levels. Our findings suggest a unique dyslipidemia profile in Chinese women with EBC treated with endocrine therapy.

This study has some limitations. The research participants were from a single-center cohort of the West China Hospital, Sichuan University, Western China, which does not represent the general population in China. This study lacked a control group (i.e., BC patients who did not receive hormonal therapy) and did not assess some confounders (i.e., diet and physical activity). This study focused on investigating the incidence of dyslipidemia and did not explore the incidence of ASCVD or CVD.

## Conclusion

This study was based on the analysis of a large-sample BC cohort in China. Among the hormone receptor-positive EBC patients with normal blood lipid levels, dyslipidemia occurred in one-third of patients within 5 years after receiving endocrine therapy. Importantly, the 5-year incidence of dyslipidemia was close to one-third among premenopausal patients in China. However, clinicians have rarely considered the impact of endocrine drugs on lipidemia when choosing the endocrine drugs for premenopausal BC patients. The incidence of abnormal TC and LDL-C levels was higher in the AI group than that in the SERM group in both postmenopausal and premenopausal patients. Therefore, AI is more likely to cause abnormal TC and LDL-C levels than SERM. In summary, dyslipidemia is an extremely common event among premenopausal and postmenopausal women and must be closely detected in those with BC.

## Data availability statement

The datasets presented in this article are not readily available because of data security protection, the dataset used and analyzed in the current research can be applied to the corresponding author according to reasonable requirements, and the analysis must be performed locally after passing the application. Requests to access the datasets should be directed to zhongxiaorongzxr@163.com.

## Ethics statement

The studies involving human participants were reviewed and approved by the biomedical research ethics committee of West China Hospital (reference number: 20200427). The patients/participants provided their written informed consent to participate in this study.

## Author contributions

XZ and TL were responsible for the conception and design of the study. JW, JY, JQ, JJ, and YH were responsible for the data and project management. JW, JY, JQ, and JJ performed data cleaning and analysis. JW, XZ, KZ, and TL were responsible for the interpretation of data. JW, JY, JQ, JJ, YH, HZ, KZ, TL, and XZ drafted and revised the manuscript. All authors approved the final manuscript as submitted and agreed to be accountable for all aspects of the work.

## Funding

This work was supported by the National Key Development Plan for Precision Medicine Research (grant number: 2017YFC0910004) and 135 projects for disciplines of excellence, West China Hospital, Sichuan University (grant numbers: ZYGD18012 and ZYJC21035).

## Conflict of interest

The authors declare that the research was conducted in the absence of any commercial or financial relationships that could be construed as a potential conflict of interest.

## Publisher’s note

All claims expressed in this article are solely those of the authors and do not necessarily represent those of their affiliated organizations, or those of the publisher, the editors and the reviewers. Any product that may be evaluated in this article, or claim that may be made by its manufacturer, is not guaranteed or endorsed by the publisher.
