# Supplementary material for: Comparison of dyslipidemia incidence in Chinese early-stage breast cancer patients following different endocrine therapies: A population-based cohort study
Source: Front Endocrinol (Lausanne). 2022 Sep 6;13:815960. doi: 10.3389/fendo.2022.815960 (PMC9486544; doi:10.3389/fendo.2022.815960)
Supplement: Supplementary file 1 [file Table_1.docx]

**Figure**

**

**

Supplementary Figure 1 **Cumulative abnormal TG and HDL-C incidence among early breast cancer pre-menopause patients for different endocrine therapy**

Cumulative curves showing TG (A and C) and HDL-C (B and D) dyslipidemia incidence. P values were obtained from log-rank tests for comparisons of abnormal TG and HDL-C incidence between different endocrine therapy groups. SERM, selective estrogen receptor modulator; TAM, tamoxifen; TOR, Toremifene; AI, aromatase inhibitor; ANA, Anastrozole; LET, Letrozole; EXE, Exemestane; OFS, ovarian function suppression; TG, Triglyceride; HDL-C, HDL cholesterol.





Supplementary Figure 2 **Cumulative abnormal TG and HDL-C incidence among early breast cancer menopause patients for different endocrine therapy**

Cumulative curves showing TG (A and C) and HDL-C (B and D) dyslipidemia incidence. P values were obtained from log-rank tests for comparisons of abnormal TG and HDL-C incidence between different endocrine therapy groups. SERM, selective estrogen receptor modulator; TAM, tamoxifen; TOR, Toremifene; AI, aromatase inhibitor; ANA, Anastrozole; LET, Letrozole; EXE, Exemestane; TG, Triglyceride; HDL-C, HDL cholesterol.

| **Supplementary Table 1 A Comparison of TC and TG dyslipidemia among premenopause early breast cancer patients within five-years** | | | | | | | | | | | | | |
| --- | --- | --- | --- | --- | --- | --- | --- | --- | --- | --- | --- | --- | --- |
|  | **TC** | | | | | | **TG** | | | | | | |
| **Treatment** | **No. of**  **event** | **Duration,**  **Median [range]** | **Incidence within 1 year [95%CI]** | **P** | **Incidence within 5 years [95%CI]** | **P** | | **No. of**  **event** | **Duration,**  **Median [range]** | **Incidence within 1**  **year [95%CI]** | **P** | **Incidence within 5**  **years [95%CI]** | P |
| OFS+AI (n=189) | **24** | 8.47 [1.35, 56.42] | 8.2% [4%, 12.2%] | <0.001 | 21.5% [11.9%, 30.1%] | <0.001 | | **34** | 4.31 [1.06, 33.71] | 15.1% [9.5%, 20.2%] | 0.29 | 23.4% [15.5%, 30.5%] | 0.23 |
| OFS+SERM (n=89) | **0** | - | - |  | - |  |  | **10** | 7.74 [1.55, 21.39] | 9.4% [ 3%, 15.4%] |  | 12.4% [ 4.8%, 19.4%] |  |
| SERM (n=980) | **17** | 5.13 [3.19, 42.77] | 1.1% [1.8%, 0.4%] |  | 2.4% [3.5%, 1.2%] |  |  | **173** | 8.00 [1.03, 57.00] | 12.2% [10.0%, 14.3%] |  | 25.3% [21.4%, 29%] |  |
| **Drug** | **No. of**  **event** | **Duration,**  **Median [range]** | **Incidence within 1 year [95%CI]** | **P** | **Incidence within 5 years [95%CI]** | **P** | | **No. of**  **event** | **Duration,**  **Median [range]** | **Incidence within 1**  **year [95%CI]** | **P** | **Incidence within 5**  **years [95%CI]** | **P** |
| OFS+ANA (n=97) | **12** | 16.71 [2.39, 53.23] | 5.8% [0.7%, 10.6%] | <0.001 | 30.8% [9.5%, 47.1%] | <0.001 | | **16** | 3.92 [1.06, 33.71] | 14.7% [7.0%, 21.8%] | 0.40 | 23.4% [ 10.5%, 34.5%] | 0.43 |
| OFS+LET (n=67) | **11** | 4.61 [1.35, 56.42] | 14.8% [ 5.3%, 23.3%] |  | 20.9% [ 8.2%, 31.9%] |  |  | **14** | 9.03 [1.35, 27.74] | 14.8% [5.3%, 23.4%] |  | 26.4% [12.9%, 37.8%] |  |
| OFS+EXE (n=25) | **1** | 16.58[16.58, 16.58] | - |  | 4.5% [ 0%, 12.9%] |  |  | **4** | 2.90 [1.23, 7.39] | 16.7% [0.3%, 30.3%] |  | 16.7% [0.3%, 30.3%] |  |
| OFS+TAM (n=87) | **0** | - | - |  | - |  |  | **9** | 9.19 [1.55, 21.39] | 8.5% [2.3%, 14.3%] |  | 11.6% [ 4.1%, 18.5%] |  |
| TOR (n=40) | **0** | - | - |  | - |  |  | **5** | 7.95 [1.03, 57.00] | 10.9% [0.2%, 20.5%] |  | 13.9% [1.8%, 24.6%] |  |
| TAM (n=940) | **17** | 5.13 [3.19, 42.77] | 1.1% [0.4%, 1.8%] |  | 2.5% [1.2%, 3.7%] |  |  | **168** | 10.81 [2.55, 18.61] | 12.2% [10.0%, 14.4%] |  | 25.7% [21.7%, 29.6%] |  |

SERM, selective estrogen receptor modulator; TAM, tamoxifen; TOR, Toremifene; AI, aromatase inhibitor; ANA, Anastrozole; LET, Letrozole; EXE, Exemestane; OFS, ovarian function suppression; TC, Total cholesterol; TG, Triglyceride;

| **Supplementary Table 1 B Comparison of HDL-C and LDL-C dyslipidemia among premenopause early breast cancer patients within five-years** | | | | | | | | | | | | |
| --- | --- | --- | --- | --- | --- | --- | --- | --- | --- | --- | --- | --- |
|  | **HDL-C** | | | | | | **LDL-C** | | | | | |
| **Treatment** | **No. of**  **event** | **Duration,**  **Median [range]** | **Incidence within 1 year [95%CI]** | **P** | **Incidence within 5 years [95%CI]** | **P** | **No. of**  **event** | **Duration,**  **Median [range]** | **Incidence within 1 year [95%CI]** | **P** | **Incidence within 5**  **years [95%CI]** | P |
| OFS+AI (n=189) | **8** | 5.58 [1.06, 30.10] | 3.5% [ 0.7%, 6.1%] | 0.85 | 5.8% [ 1.5%, 9.8%] | 0.49 | **14** | 8.02 [1.23, 51.97] | 5.3% [1.8%, 8.6%] | <0.001 | 11.1% [4.6%, 17.2%] | <0.001 |
| OFS+SERM (n=89) | **6** | 7.61 [2.48, 23.19] | 5.9% [ 0.7%, 10.8%] |  | 7.6% [ 1.5%, 13.3%] |  | **0** | - | - |  | - |  |
| SERM (n=980) | **69** | 8.19 [1.48, 55.10] | 4.3% [5.6%, 3%] |  | 9.8% [12.2%, 7.3%] |  | **7** | 18.03 [1.26, 26.58] | 0.3% [0.0%, 0.7%] |  | 0.9% [0.2%, 1.6%] |  |
| **Drug** | **No. of**  **event** | **Duration,**  **Median [range]** | **Incidence within 1 year [95%CI]** | **P** | **Incidence within 5 years [95%CI]** | **P** | **No. of**  **event** | **Duration,**  **Median [range]** | **Incidence within 1 year [95%CI]** | **P** | **Incidence within 5**  **years [95%CI]** | **P** |
| OFS+ANA (n=97) | **3** | 3.77 [1.06, 7.84] | 3.4% [ 0.0%, 7.1%] | 0.76 | 3.4% [ 0.0%, 7.1%] | 0.33 | **9** | 9.35 [2.81, 51.97] | 6% [0.9%, 11%] | <0.001 | 18.9% [3.3%, 31.9%] | <0.001 |
| OFS+LET (n=67) | **2** | 13.58 [1.87, 25.29] | 1.5% [ 0.0%, 4.5%,] |  | 4.1% [ 0.0%, 9.6%] |  | **3** | 3.65 [1.35, 4.68] | 4.8% [ 0.0%, 9.9%] |  | 4.8% [ 0.0%, 9.9%] |  |
| OFS+EXE (n=25) | **3** | 7.39 [1.23, 30.10] | 8.5% [ 0.0%, 19.2%] |  | 17.7% [ 0.0%, 35.3%] |  | **2** | 8.90 [1.23, 16.58] | 4.2% [ 0.0%, 11.8%] |  | 8.7% [ 0.0%, 19.6%] |  |
| OFS+TAM (n=87) | **5** | 9.19 [2.84, 23.19] | 4.9% [ 0.1%, 9.4%] |  | 6.6% [ 0.8%, 12.0%] |  | **0** | - | - |  | - |  |
| TOR (n=40) | **1** | 2.55 [2.55, 2.55] | 2.6% [ 0.0%, 7.4%] |  | 2.6% [ 0%, 7.4%] |  | **0** | - | - |  | - |  |
| TAM (n=940) | **68** | 8.27 [1.48, 55.10] | 4.4% [3.0%, 5.7%] |  | 10.1% [7.5%, 12.5%] |  | **7** | 18.03 [1.26, 26.58] | 0.3% [0.0%, 0.7%] |  | 1% [0.2%, 1.7%] |  |

SERM, selective estrogen receptor modulator; TAM, tamoxifen; TOR, Toremifene; AI, aromatase inhibitor; ANA, Anastrozole; LET, Letrozole; EXE, Exemestane; OFS, ovarian function suppression; HDL-C, HDL cholesterol; LDL-C, LDL cholesterol;

| **Supplementary Table 2 A Comparison of TC and TG dyslipidemia among menopause early breast cancer patients within five-years** | | | | | | | | | | | | | | |
| --- | --- | --- | --- | --- | --- | --- | --- | --- | --- | --- | --- | --- | --- | --- |
|  | | **TC** | | | | | | **TG** | | | | | | |
| **Treatment** | **No. of**  **event** | | **Duration,**  **Median [range]** | **Incidence within 1 year [95%CI]** | **P** | **Incidence within 5 years [95%CI]** | **P** | | **No. of**  **event** | **Duration,**  **Median [range]** | **Incidence within 1**  **year [95%CI]** | **P** | **Incidence within 5**  **years [95%CI]** | P |
| AI (n=461) | **77** | | 7.61 [1.32, 59.23] | 13.3% [10.0%, 16.5%] | 0.003 | 22% [17.0%, 26.6%] | <0.001 | | **80** | 5.85 [1.10, 44.16] | 13.4% [10.1%, 16.6%] | 0.48 | 22.1% [17.4%, 26.5%] | 0.45 |
| SERM (n=164) | **7** | | 9.42 [3.68, 53.65] | 2.8% [0.1%, 5.5] |  | 8.8% [1.3%, 15.7] |  |  | **32** | 5.94 [1.19, 48.45] | 14.8% [8.9%, 20.4] |  | 25.4% [16.6%, 33.4%] |  |
| **Drug** | **No. of**  **event** | | **Duration,**  **Median [range]** | **Incidence within 1 year [95%CI]** | **P** | **Incidence within 5 years [95%CI]** | **P** | | **No. of**  **event** | **Duration,**  **Median [range]** | **Incidence within 1**  **year [95%CI]** | **P** | **Incidence within 5**  **years [95%CI]** | **P** |
| ANA (n=239) | **47** | | 7.61 [1.32, 59.23] | 14.2% [9.4%, 18.7%] | 0.027 | 27.8% [19.3%, 35.4%] | <0.001 | | **43** | 4.84 [1.29, 44.16] | 14.7% [9.9%, 19.2%] | 0.77 | 22.2% [15.7%, 28.3%] | 0.60 |
| TAM (n=157) | **6** | | 7.69 [3.68, 48.45] | 2.9% [0.1%, 5.7%] |  | 6.6% [0.6%, 12.3%] |  |  | **31** | 5.97 [1.19, 48.45] | 14.8% [8.7%, 20.4%] |  | 25.9% [16.7%, 34.2%] |  |
| LET (n=186) | **26** | | 8.16 [1.87, 31.29] | 12.5% [7.2%, 17.4%] |  | 17.4% [10.9%, 23.4%] |  |  | **33** | 7.45 [1.10, 43.32] | 12.6% [7.4%, 17.5%] |  | 23.4% [15.6%, 30.4%] |  |
| EXE (n=36) | **4** | | 3.53 [2.65, 11.45] | 12.0% [ 0.2%, 22.4%] |  | 12.0% [ 0.2%, 22.4%] |  |  | **4** | 6.76 [2.74, 34.16] | 9.0% [ 0.0%, 18.3%] |  | 14.4% [ 0.0%, 27.1%] |  |

SERM, selective estrogen receptor modulator; TAM, tamoxifen; TOR, Toremifene; AI, aromatase inhibitor; ANA, Anastrozole; LET, Letrozole; EXE, Exemestane; OFS, ovarian function suppression; TC, Total cholesterol; TG, Triglyceride;

| **Supplementary Table 2 B Comparison of HDL-C and LDL-C dyslipidemia among menopause early breast cancer patients within five-years** | | | | | | | | | | | | |
| --- | --- | --- | --- | --- | --- | --- | --- | --- | --- | --- | --- | --- |
|  | **HDL-C** | | | | | | **LDL-C** | | | | | |
| **Treatment** | **No. of**  **event** | **Duration,**  **Median [range]** | **Incidence within 1 year [95%CI]** | **P** | **Incidence within 5 years [95%CI]** | **P** | **No. of**  **event** | **Duration,**  **Median [range]** | **Incidence within 1**  **year [95%CI]** | **P** | **Incidence within 5**  **years [95%CI]** | P |
| AI (n=461) | **37** | 7.77 [1.06, 58.55] | 5.2% [3.0%, 7.3] | 0.71 | 12.6% [8.1%, 16.9] | 0.71 | **46** | 7.24 [1.29, 59.23] | 7.9% [5.3%, 10.5%] | 0.045 | 13.3% [9.3%, 17.2%] | <0.001 |
| SERM (n=164) | **11** | 5.90 [1.39, 18.87] | 6.2% [2.2%, 10.1%] |  | 7.9% [3.3%, 12.4%] |  | **0** | - | 0% [0%, 0%] |  | 0% [0%, 0%] |  |
| **Drug** | **No. of**  **event** | **Duration,**  **Median [range]** | **Incidence within 1 year [95%CI]** | **P** | **Incidence within 5 years [95%CI]** | **P** | **No. of**  **event** | **Duration,**  **Median [range]** | **Incidence within 1**  **year [95%CI]** | **P** | **Incidence within 5**  **years [95%CI]** | **P** |
| ANA (n=239) | **21** | 9.23 [1.29, 58.55] | 4.9% [2.0%, 7.8] | 0.85 | 16% [8.1%, 23.3%] | 0.76 | **27** | 7.68 [1.29, 59.23] | 7.8% [4.2%, 11.3%] | 0.19 | 16.7% [9.6%, 23.3] | <0.001 |
| TAM (n=157) | **11** | 5.90 [1.39, 18.87] | 6.5% [2.3%, 10.5%] |  | 8.2% [3.4%, 12.8%] |  | **0** | - | - |  | - |  |
| LET (n=186) | **12** | 6.66 [1.42, 54.77] | 5.4% [1.9%, 8.8%] |  | 8.8% [3.3%, 13.9] |  | **16** | 7.24 [1.87, 31.29] | 8% [3.7%, 12.2%] |  | 10.5% [5.4%, 15.4%] |  |
| EXE (n=36) | **4** | 12.34 [1.06, 34.16] | 5.8% [ 0.0%, 13.3%] |  | 14.6% [ 0.0%, 27.5%] |  | **3** | 2.74 [2.65, 4.32] | 8.8% [ 0.0%, 17.8%] |  | 8.8% [ 0.0%, 17.8%] |  |

SERM, selective estrogen receptor modulator; TAM, tamoxifen; TOR, Toremifene; AI, aromatase inhibitor; ANA, Anastrozole; LET, Letrozole; EXE, Exemestane; OFS, ovarian function suppression; HDL-C, HDL cholesterol; LDL-C, LDL cholesterol;

| **Supplementary Table 3** Pairwise comparisons among pre-menopause patients  adjusted by Benjamini-Hochberg procedure | | | | | | | |
| --- | --- | --- | --- | --- | --- | --- | --- |
|  |  | OFS+AI | | | OFS+SERM | |  |
|  |  | 12 months | 60 months | 12 months | | 60 months |  |
| TC | OFS+SERM | **0.019** | **0.001** | - | | - |  |
|  | SERM | **0.001** | **<0.001** | 0.194 | | 0.207 |  |
| TG | OFS+SERM | 0.349 | 0.220 | - | | - |  |
|  | SERM | 0.349 | 0.442 | 0.378 | | 0.220 |  |
| HDL-C | OFS+SERM | 0.985 | 0.74 | - | | - |  |
|  | SERM | 0.985 | 0.709 | 0.985 | | 0.922 |  |
| LDL-C | OFS+SERM | **0.050** | **0.011** | - | | - |  |
|  | SERM | **<0.001** | **<0.001** | 0.390 | | 0.416 |  |

TC, Total cholesterol; TG, Triglyceride; HDL-C, HDL cholesterol; LDL-C, LDL cholesterol;

The bold font represents the drugs group for further analysis, followed the selection criteria as: 1. p-value is less than 0.05; 2. Incidence of interest events is not very few.

| **Supplementary Table 4** Pairwise comparisons among menopause patients adjusted by Benjamini-Hochberg procedure | | | |
| --- | --- | --- | --- |
|  |  | AI | |
|  |  | 12 months | 60 months |
| TC | SERM | **0.002** | **<0.001** |
| TG | SERM | 0.480 | 0.451 |
| HDL-C | SERM | 0.712 | 0.714 |
| LDL-C | SERM | **0.045** | **<0.001** |

TC, Total cholesterol; TG, Triglyceride; HDL-C, HDL cholesterol; LDL-C, LDL cholesterol;

The bold font represents the drugs group for further analysis, followed the selection criteria as: 1. p-value is less than 0.05; 2. Incidence of interest events is not very few.

| **Supplementary Table 5** Pairwise comparisons among pre-menopause patients  adjusted by Benjamini-Hochberg procedure | | | | | | | | | | | | | | | | | | |  |
| --- | --- | --- | --- | --- | --- | --- | --- | --- | --- | --- | --- | --- | --- | --- | --- | --- | --- | --- | --- |
|  |  | OFS+ANA | | OFS+EXE | | | OFS+LET | | | | OFS+TAM | | | | TAM | | | |  |
|  |  | 12 m | 60 m | 12 m | 60 m | | 12 m | | 60 m | | 12 m | | 60 m | | 12 m | | 60 m | |  |
| TC | OFS+EXE | 0.375 | 0.226 | - | - | | - | | - | | - | | - | | - | | - | |  |
|  | OFS+LET | 0.180 | 0.864 | 0.182 | 0.226 | | - | | - | | - | | - | | - | | - | |  |
|  | OFS+TAM | 0.180 | **0.001** | 1.000 | 0.16 | | 0.005 | | **<0.001** | | - | | - | | - | | - | |  |
|  | TAM | 0.202 | **<0.001** | 0.595 | 0.452 | | <0.001 | | **<0.001** | | 0.359 | | 0.304 | | - | | - | |  |
|  | TOR | 0.877 | **0.02** | 0.359 | 0.311 | | 0.359 | | **0.02** | | 0.170 | | 1 | | 0.222 | | 0.452 | |  |
| TG | OFS+EXE | 0.846 | 0.918 | - | - | | - | | - | | - | | - | | - | | - | |  |
|  | OFS+LET | 0.670 | 0.854 | 0.875 | 0.854 | | - | | - | | - | | - | | - | | - | |  |
|  | OFS+TAM | 0.737 | 0.549 | 0.670 | 0.744 | | 0.577 | | 0.549 | | - | | - | | - | | - | |  |
|  | TAM | 0.996 | 0.854 | 0.845 | 0.918 | | 0.664 | | 0.744 | | 0.670 | | 0.549 | | - | | - | |  |
|  | TOR | 0.875 | 0.744 | 0.845 | 0.854 | | 0.670 | | 0.744 | | 0.875 | | 0.854 | | 0.875 | | 0.744 | |  |
| HDL-C | OFS+EXE | 0.816 | 0.556 | - | - | | - | | - | | - | | - | | - | | - | |  |
|  | OFS+LET | 0.910 | 0.908 | 0.816 | 0.556 | | - | | - | | - | | - | | - | | - | |  |
|  | OFS+TAM | 0.970 | 0.661 | 0.816 | 0.556 | | 0.910 | | 0.661 | | - | | - | | - | | - | |  |
|  | TAM | 0.910 | 0.556 | 0.816 | 0.606 | | 0.816 | | 0.556 | | 0.910 | | 0.755 | | - | | - | |  |
|  | TOR | 0.915 | 0.908 | 0.816 | 0.556 | | 0.910 | | 0.908 | | 0.915 | | 0.661 | | 0.910 | | 0.556 | |  |
| LDL-C | OFS+EXE | 0.836 | 0.726 | - | - | | - | | - | | - | | - | | - | | - | |  |
|  | OFS+LET | 0.584 | 0.265 | 0.844 | 0.66 | | - | | - | | - | | - | | - | | - | |  |
|  | OFS+TAM | 0.078 | **0.008** | 0.122 | **0.025** | | 0.188 | | 0.089 | | - | | - | | - | | - | |  |
|  | TAM | **<0.001** | **<0.001** | 0.119 | | **0.001** | | 0.115 | | **0.008** | | 0.678 | | 0.56 | | - | | - | |
|  | TOR | 0.844 | 0.072 | 0.853 | 0.143 | | 0.785 | | 0.265 | | 0.115 | | 1 | | **0.016** | | 0.668 | |  |

TC, Total cholesterol; TG, Triglyceride; HDL-C, HDL cholesterol; LDL-C, LDL cholesterol;

The bold font represents the drugs group for further analysis, followed the selection criteria as: 1. p-value is less than 0.05; 2. Incidence of interest events is not very few.

| **Supplementary Table 6** Pairwise comparisons among menopause patients adjusted by Benjamini-Hochberg procedure | | | | | | | |  |
| --- | --- | --- | --- | --- | --- | --- | --- | --- |
|  |  |  | ANA |  | TAM |  | LET | |
|  |  | 12 m | 60 m | 12 m | 60 m | 12 m | 60 m | |
| TC | TAM | **0.016** | **<0.001** | - | - | - | - | |
|  | LET | 0.718 | 0.199 | **0.024** | **0.004** | - | - | |
|  | EXE | 0.718 | 0.231 | 0.365 | 0.231 | 0.718 | 0.609 | |
| TG | TAM | 0.849 | 0.758 | - | - | - | - | |
|  | LET | 0.849 | 0.891 | 0.849 | 0.758 | - | - | |
|  | EXE | 0.810 | 0.561 | 0.810 | 0.561 | 0.733 | 0.561 | |
| HDL-C | TAM | 0.868 | 0.821 | - | - | - | - | |
|  | LET | 0.868 | 0.821 | 0.873 | 0.821 | - | - | |
|  | EXE | 0.868 | 0.821 | 0.835 | 0.821 | 0.832 | 0.821 | |
| LDL-C | TAM | 0.389 | **<0.001** | - | - | - | - | |
|  | LET | 0.528 | 0.608 | 0.168 | **<0.001** | - | - | |
|  | EXE | 0.893 | 0.659 | 0.473 | **0.001** | 0.872 | 0.934 | |

TC, Total cholesterol; TG, Triglyceride; HDL-C, HDL cholesterol; LDL-C, LDL cholesterol;

The bold font represents the drugs group for further analysis, followed the selection criteria as: 1. p-value is less than 0.05; 2. Incidence of interest events is not very few.
